# Statins affect human glioblastoma and other cancers through TGF-β inhibition

**DOI:** 10.18632/oncotarget.26733

**Published:** 2019-03-01

**Authors:** Aizhen Xiao, Breanna Brenneman, Desiree Floyd, Laurey Comeau, Kelsey Spurio, Inan Olmez, Jeongwu Lee, Ichiro Nakano, Jakub Godlewski, Agnieszka Bronisz, Noritaka Kagaya, Kazuo Shin-ya, Benjamin Purow

**Affiliations:** ^1^ Departments of Neurology, University of Virginia, Charlottesville, VA 22908, USA; ^2^ Biochemistry and Molecular Genetics, University of Virginia, Charlottesville, VA 22908, USA; ^3^ Department of Stem Cell Biology and Regenerative Medicine, Lerner Research Institute, Cleveland Clinic, Cleveland, OH 44195, USA; ^4^ Department of Neurosurgery, University of Alabama, Birmingham, AL 35233, USA; ^5^ Department of Neurosurgery, Brigham and Women's Hospital, Boston, MA 02115, USA; ^6^ National Institute of Advanced Industrial Science and Technology (AIST), Tokyo 135-0064, Japan

**Keywords:** statin, glioblastoma, TGF-beta, SMAD3, RhoA/ROCK

## Abstract

The cholesterol-lowering statins have known anti-cancer effects, but the mechanisms and how to utilize statins in oncology have been unclear. We noted in the CellMiner database that statin activity against cancer lines correlated with higher expression of TGF-β target genes such as *SERPINE1* and *ZYX*. This prompted us to assess whether statins affected TGF-β activity in glioblastoma (GBM), a cancer strongly influenced by TGF-β and in dire need of new therapeutic approaches. We noted that statins reduced TGF-β activity, cell viability and invasiveness, Rho/ROCK activity, phosphorylation and activity of the TGF-β mediator Smad3, and expression of TGF-β targets ZYX and SERPINE1 in GBM and GBM-initiating cell (GIC) lines. Statins were most potent against GBM, GIC, and other cancer cells with high TGF-β activity, and exogenous TGF-β further sensitized mesenchymal GICs to statins. Statin toxicity was rescued by addition of exogenous mevalonolactone or geranylgeranyl pyrophosphate, indicating that the observed effects reflected inhibition of HMG CoA-reductase by the statins. Simvastatin significantly inhibited the growth of subcutaneous GIC grafts and prolonged survival in GIC intracranially grafted mice. These results indicate where the statins might best be applied as adjunct therapies in oncology, against GBM and other cancers with high TGF-β activity, and have implications for other statin roles outside of oncology.

## INTRODUCTION

The statins inhibit the HMG-CoA reductase enzyme and are among the most widely-prescribed drugs in the world for their cholesterol-lowering function. However, an additional effect on cancers has long been suspected, based in part on numerous studies that have shown decreased risk of death from various cancers in patients on statins. This remains unclear for glioblastoma (GBM), however [1]. A few potential mechanisms for statin effects on cancer cells have been posited, including down-regulation of NF-κB, but no central mechanism has emerged [2]. Though prior reports have shown that statins can have anti-glioma effects and have linked them to components of the Transforming Growth Factor-beta (TGF-β) pathway [3–10], the statins have not previously been shown to potently inhibit TGF-β as their primary anti-cancer mechanism.

In normal cells, TGF-β is an inflammatory pathway that drives expression of p21 and other tumor suppressors and acts to curb the cell cycle. However, in cancer cells TGF-β drives malignant behaviors and is a promising therapeutic target [11, 12]. It stimulates epithelial-mesenchymal transition, fostering invasion, metastasis, and possibly treatment resistance. Local TGF-β production strongly suppresses anti-tumor immunity [13]. TGF-β signaling also promotes cancer cell survival. It may be an especially promising target for the treatment-resistant and tumorigenic GBM-initiating cell (GIC) subpopulation [14–16], and for the mesenchymal GBM subtype [17]. Unfortunately, targeting TGF-β signaling, and the mesenchymal cancers with elevated activity, has been hampered by the absence of TGF-β inhibitors in the clinic.

We noted a potential link between the statins and TGF-β in the CellMiner database. This database makes available complete profiling data for the NCI-60 panel of cancer cell lines including gene expression, microRNA expression, and sensitivity to a >20,000 compound library [18]. Online tools enable pattern comparisons across the datasets (http://discover.nci.nih.gov/cellminer/analysis.do), and using these tools we noted that cancer cell line sensitivity to several statins in the compound library correlated with the expression of certain TGF-β target genes (such as *SERPINE1* and *ZYX*) and mesenchymal genes [19, 20]. This suggested that TGF-β activity might sensitize to the statins, as well as hinting that the statins could be functioning as TGF-β inhibitors. We therefore evaluated in GBM, a cancer in dire need of new therapeutic approaches and in which TGF-β plays an oncogenic role, whether the statins interacted with TGF-β activity at physiologically relevant concentrations. This work is the first to show potent TGF-β inhibition by the statins in GBM and other cancers, and further indicates that TGF-β inhibition is the key mechanism for direct anti-cancer activity of the statins.

## RESULTS

### Statin activity in a large panel of cancer cell lines correlates with expression of TGF-β target genes

Using the Pattern Comparison function in the CellMiner database [18, 21], we noted that cancer cell line sensitivity to several statins in the compound library correlated strikingly with expression of TGF-β target genes *SERPINE1* and *ZYX* (Table 1) [19, 20]. All of the statins in the compound library were found among those which decreased cancer cell viability with a pattern correlating significantly with expression of these TGF-β target genes (p-values were 1.9×10^-12^ for *SERPINE1* and 7.7×10^-16^ for *ZYX* using hypergeometric tests to verify enrichment). This suggested that TGF-β activity might sensitize to the statins, and that the statins could be functioning as TGF-β inhibitors.

**Table 1:** Statin effects on cancer cell viability correlate with expression of TGF-β target genes SERPINE1 and ZYX in the CellMiner database

TGF-β target Gene	Statin # with significant correlation with effect	Compound # with significant correlation with effect	Total # statins tested in CellMiner library	Total # compounds tested CellMiner library	P value
SERPINE1	7	436	7	20515	1.9 × 10^−12^
ZYX	8	261	8	19952	7.7 × 10^−16^

### Statins have widely varying toxicity to glioblastoma and other cancer cells

**Figure 1:**
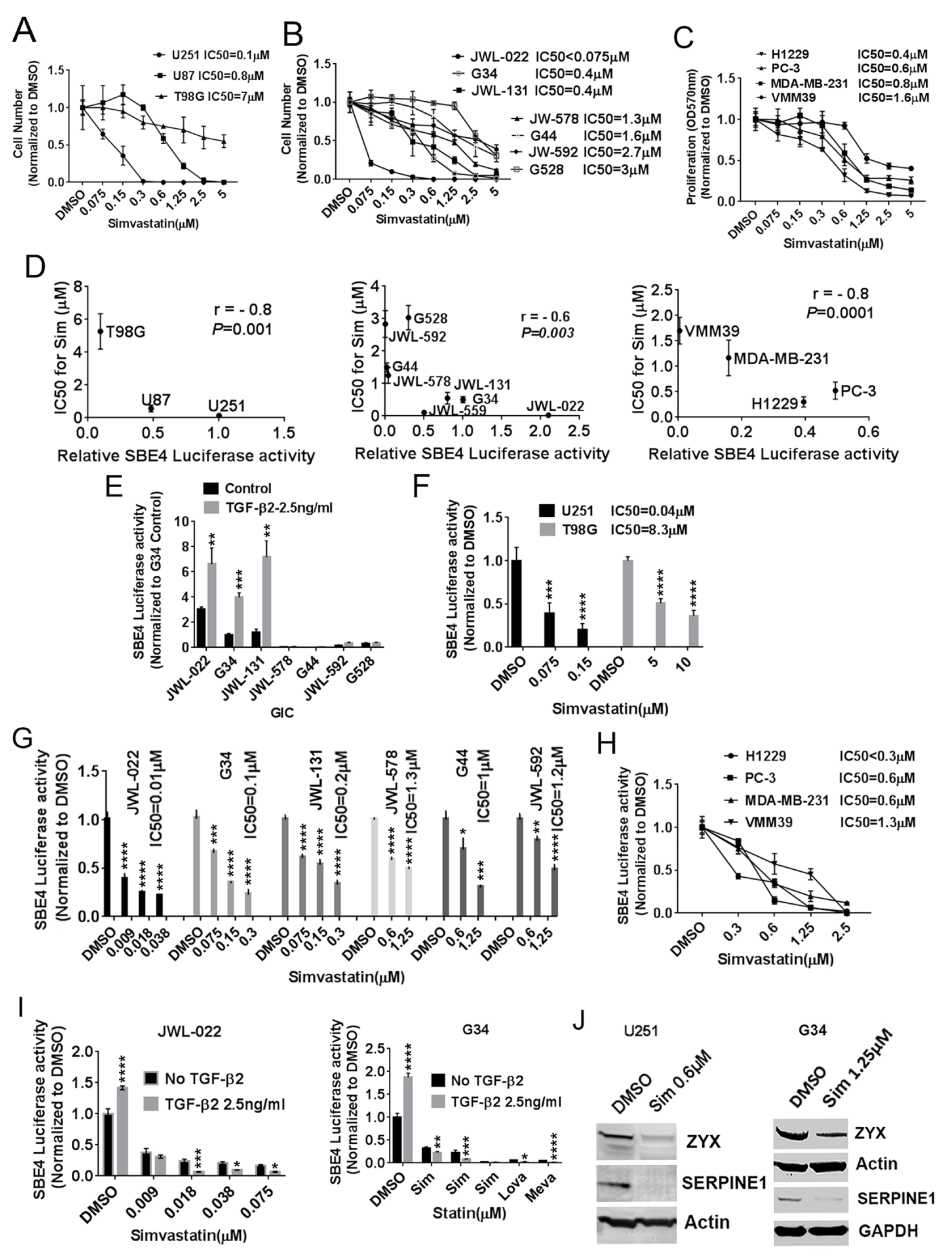
Statins impair the viability of GBM and other cancer cells largely through TGF-β inhibition **(A)** IC50 (μM) values of simvastatin for U251MG, U87MG, and T98G GBM cells with 1%FBS-MEM medium for 6 days. **(B)** IC50 (μM) values of simvastatin for GICs. **(C)** IC50s (μM) of simvastatin-treated lung cancer H1229, prostate cancer PC-3, breast cancer MDA-MB-231 and melanoma VMM39. Cell proliferation measured at OD570nm. **(D)** IC50 for simvastatin demonstrates a significant inverse correlation with normalized SBE4-luciferase activity in GBM lines (left panel), seven GIC lines (middle panel) and four other cancers (right panel). **(E)** SBE4 activity +/- exogenous TGF-β in seven GICs. **(F)** Simvastatin lowered SBE4-luciferase activity at physiologically relevant concentrations (IC50 0.04μM) in high-TGF-β activity U251. **(G)** Simvastatin lowered SBE4-luciferase activity in six GIC lines. **(H)** Simvastatin inhibited SBE4-luciferase activity in a dose-dependent fashion in melanoma, breast, prostate, and lung cancer lines. **(I)** Simvastatin, lovastatin, and mevastatin reduced SBE4-luciferase activity in two high TGF-β activity GIC lines JWL-022 and G34, and exogenous TGF-β sensitized the effect. **(J)** Simvastatin reduced expression of the TGF-β target proteins ZYX and SERPINE1 in U251MG and G34 cells. Sim: simvastatin. Lova: lovastatin. Meva: mevastatin. All values are the mean±SD of three experiments. ^*^, *P*<0.05; ^**^, *P*<0.01. ^***^, *P*<0.001. ^****^, *P*<0.0001.

Given the important roles of TGF-β in GBM and previous work on the statins in GBM, we focused on this cancer initially. We first tested the activity of simvastatin with single dosing against widely-used GBM cell lines, noting almost a two-log variation in IC50 based on cell count across U251MG, U87MG, and T98G (Figure 1A). A similar range in effects was found in soft agar clonogenicity assays in these cells (Supplementary Figure 1A). To test whether statins other than simvastatin affected GBM cells similarly, we found that mevastatin, lovastatin, and fluvastatin had comparable effects on GBM cell viability (Supplementary Figure 1B-1D). We also assessed whether statins could affect GICs that are more representative of the disease in patients—lines which tend to be more resistant to most therapies. GIC viability was also varyingly sensitive to statins at physiologically-relevant concentrations with single dosing, with simvastatin IC50s ranging from less than 0.075 μM to 3 μM (Figure 1B). Mevastatin and lovastatin also had comparable potency to simvastatin against a GIC line (Supplementary Figure 1E). Simvastatin had moderate potency—similar to that against GICs—in viability assays of lung cancer (H1299), prostate (PC-3), breast (MDA-MB-231), and melanoma (VMM39) cell lines (Figure 1C). Commercially available TGF-β inhibitors, TGF-β RI kinase inhibitor V and LY2109761, also showed some toxicity to GBM cells and the G34 GIC line (Supplementary Figure 1F and 1G). Notably, a screen of a natural compound library for a compound selectively toxic to a mesenchymal GIC line yielded a statin compound (Supplementary Figure 2).

### Statin effects correlate inversely with and inhibit canonical TGF-β activity as indicated by a Smad3-based reporter

As the results above indicated that statins anti-cancer effects were highly variable and might correlate with TGF-β activity, we proceeded to test this more definitively in GBM, GICs, and other cancer cell lines. The SBE4-luciferase reporter plasmid has four SMAD-Binding Elements upstream of a constitutive promoter driving firefly luciferase expression, and it is a well-established and accurate indicator of canonical TGF-β activity through its mediator Smad3. In the three well-established GBM lines above, we identified a significant inverse correlation across the three lines between SBE4-luciferase activity and simvastatin IC50 (based on cell count) (Figure 1D, left panel, p=0.001, r= - 0.8). A similar inverse correlation between SBE4-luciferase activity and simvastatin IC50 also emerged in GIC lines (Figure 1D, middle panel, p=0.003, r= - 0.7) and other cancer lines (Figure 1D, right panel, p=0.0001, r= - 0.8). Low simvastatin IC50 (IC50<1μM) cells U251 MG, U87 MG, JWL-022, G34, JWL-131, H1299, PC-3, and MDA-MB 231 showed high SBE4-luciferase activity, while high simvastatin IC50 (IC50>1μM) cells T98G, G528, G44, JWL-578, JWL-592 and VMM39 showed low SBE4-luciferase activity. Further exogenous TGF-β markedly increased SBE4-luciferase activity in JWL-022, G34, JWL-131 GIC lines (Figure 1E). Interestingly, these three GIC lines with higher canonical TGF-β activity and greater sensitivity to simvastatin (Figure 1B and 1E) were typed by marker expression as belonging to the mesenchymal GBM subtype [22] (data not shown for JWL-131 cells).

The correlations between TGF-β activity or SBE4 luciferase activity and simvastatin sensitivity led us to hypothesize that the statins might inhibit TGF-β activity. We first tested this in established GBM lines, noting that simvastatin indeed lowered SBE4-luciferase activity in a dose-dependent fashion with IC50 ranging from 0.04μM to 8.3μM (Figure 1F). Lovastatin, mevastatin, and fluvastatin were similarly able to reduce SBE4-luciferase activity in the established U251 MG line (Supplementary Figure 3A). In GIC lines, simvastatin dose-dependently lowered SBE4-luciferase activity, with IC50s ranging from less than 0.01 μM to 1.2 μM (Figure 1G). Simvastatin inhibition of SBE4 luciferase activity also was observed in lung, prostate, breast, and melanoma cancer lines, with IC50s ranging from 0.3 μM to 1.3 μM (Figure 1H). Statin inhibition effect on SBE4-luciferase activity was more pronounced in higher SBE4-luciferase activity cells U251 MG, U87 MG, JWL-022, G34, JWL-131, H1299, PC-3 and MDA-MB 231, as well as when exogenous TGF-β was added in two higher SBE4-luciferase activity GIC lines JWL-022 and G34 (Figure 1I). Our results suggest that cell lines with higher SBE4 luciferase are more sensitive to statins. Notably, the IC50s for simvastatin's inhibitory effects on SBE4 luciferase activity occurred at physiologically relevant concentrations, as simvastatin blood levels in humans with typical dosing reach the mid- to high-nanomolar range. Further confirming the inhibitory effects of a statin on TGF-β activity, simvastatin reduced expression of the TGF-β target proteins ZYX and SERPINE1 in U251MG and a GIC line (Figure 1J). The commercially available TGF-β inhibitors, TGF-β RI kinase inhibitor V and LY 2109761, also decreased SBE-4 luciferase activity in established GBM lines and a GIC line (Supplementary Figure 3B and 3C).

### Statins act on cancer cells through RhoA and Smad3

**Figure 2:**
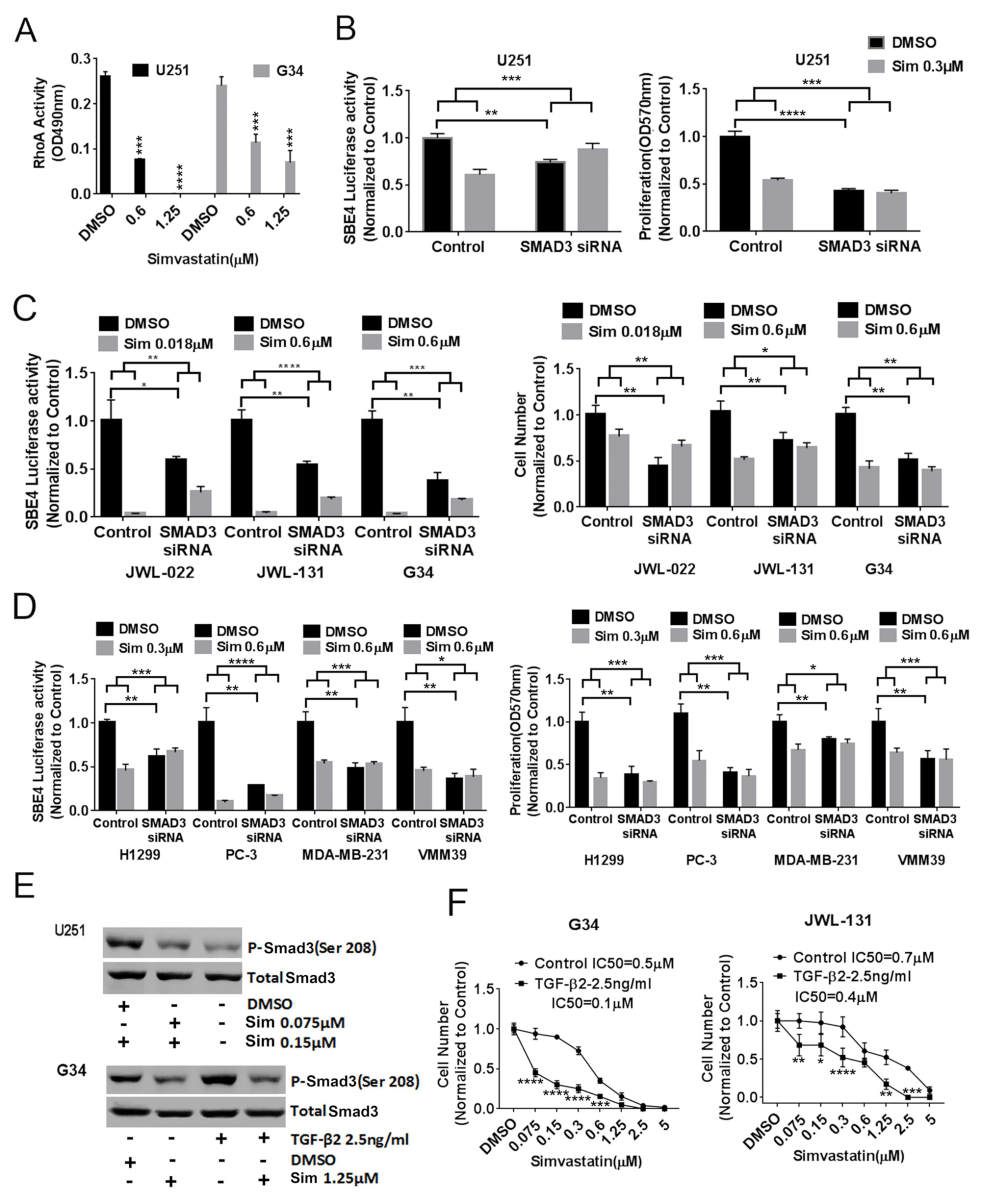
Statin acts on TGF-β through RhoA and Smad3 **(A)** Simvastatin dose-dependently decreased RhoA activity in U251MG and G34. **(B)**, **(C)**, **(D)**, *SMAD3* knockdown reduced SBE4 luciferase activity and cell proliferation and ameliorated simvastatin's effects on TGF-β reporter activity and cell proliferation in U251MG (B), in GICs JWL-022, JWL-131 and G34 (C), and in multiple other cancer types (D). **(E)** Simvastatin reduced Smad3 phosphorylation at ser208 in U251MG (top panel) and basal and exogenous TGF-β-induced Smad3 phosphorylation at ser208 in G34 (bottom panel). **(F)** Cell counts of adherent G34 and JWL-131 GICs after six days of simvastatin +/-exogenous TGF-β. Sim: simvastatin. All values are the mean±SD of three experiments. ^*^, *P*<0.05; ^**^, *P*<0.01. ^***^, *P*<0.001. ^****^, *P*<0.0001.

Statin inhibition of HMG-CoA reductase decreases farnesylation and geranylgeranylation, which in turn regulate activity of Rho and ROCK, are known to phosphorylate the canonical TGF-β mediator Smad3. We therefore hypothesized that statin effects on canonical TGF-β activity acted through this pathway. Supporting this hypothesis, we noted that simvastatin significantly decreased RhoA activity in a dose-dependent fashion in U251 MG and G34 cells (Figure 2A). To investigate whether Smad3 was indeed playing a central role in statin effects on TGF-β activity and cancer cell viability, we tested whether *SMAD3* knockdown with siRNA influenced statin effects on cancer cells. First we confirmed that *SMAD3* siRNA knockdown reduced total Smad3 protein expression in G34 cells (Supplementary Figure 3D). We observed that *SMAD3* knockdown not only significantly reduced SBE4 luciferase activity and cell numbers in U251 MG (Figure 2B), three GIC lines (Figure 2C), and four other cancer lines (Figure 2D), but also completely or partially ablated the effects of simvastatin on SBE-4 luciferase activity and cell viability in these cells (Figure 2B-2D). In addition, we noted via immunoblot that simvastatin reduced Smad3 phosphorylation at Ser-208 in U251MG (Figure 2E, top panel) and G34 cells (Figure 2E, bottom panel), sites previously shown to be phosphorylated by ROCK. We also confirmed that exogenous TGF-β increased Smad3 phosphorylation at Ser-208 (Figure 2E, bottom panel). As further evidence of the importance of TGF-β activity in the impact of statins on GBM cells, we showed that simvastatin had a greater effect on the viability in high TGF-β activity GIC lines G34 and JWL-131 when exogenous TGF-β was added in both two-dimensional growth conditions (Figure 2F) and three-dimensional growth conditions in G34 (Supplementary Figure 3E), but not in low TGF-β activity GIC lines G44 and G528 (data not shown).

### Simvastatin reduces glioblastoma migration and invasion, and induces apoptosis and autophagy

**Figure 3:**
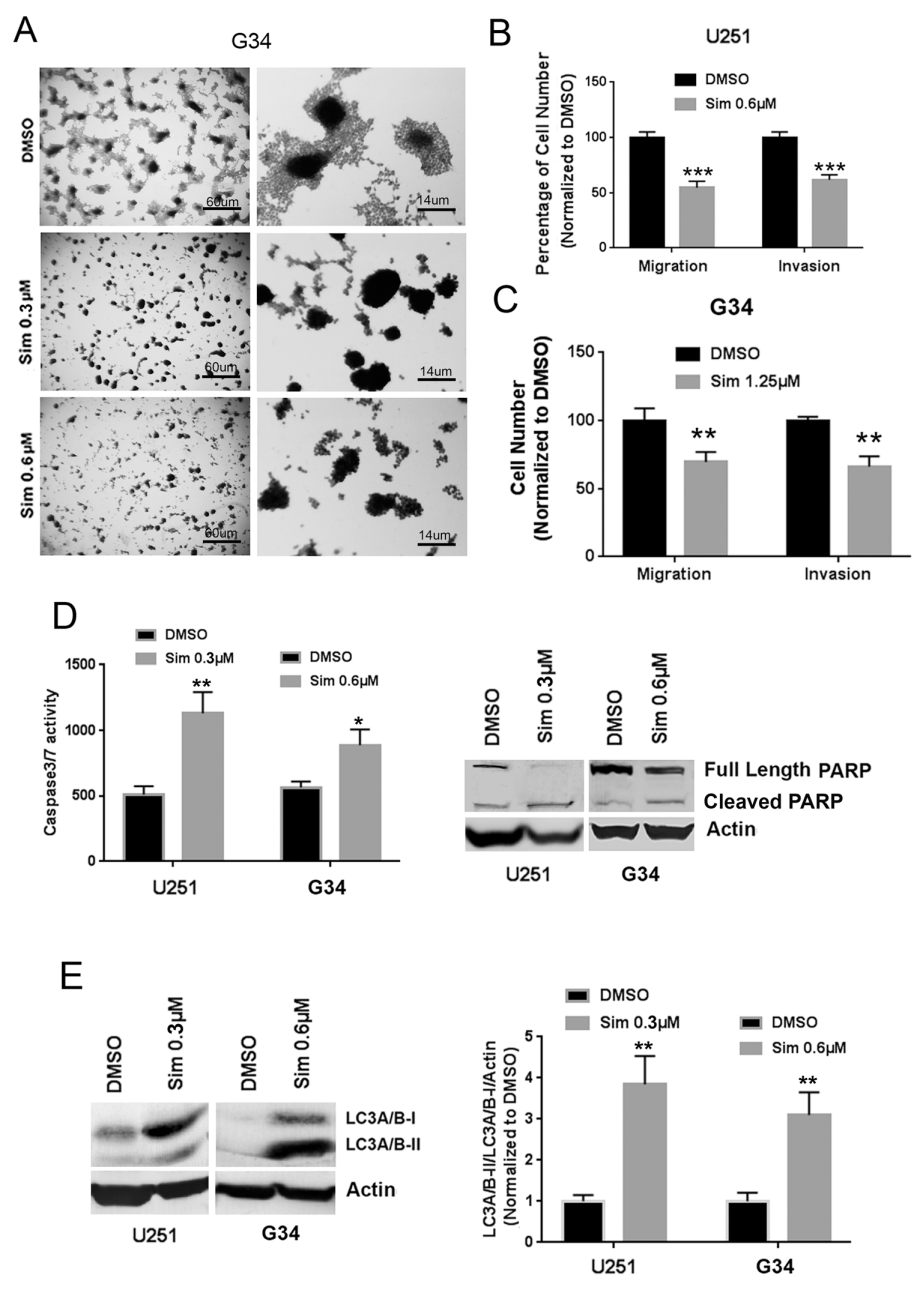
Simvastatin reduces migration and invasion and induces apoptosis and autophagy in GBM and GICs **(A)** Simvastatin ablated cell spreading from adherent spheroids of G34 with a single simvastatin dose applied for 6 days. **(B)**, **(C)** Simvastatin reduced migration and invasion of U251MG (B) and G34 cells (C). **(D)** Simvastatin induced apoptosis as indicated by increasing caspase3/7 activity (left panel) and cleaved PARP expression (right panel) in U251MG and G34. **(E)** Simvastatin stimulated autophagy as indicated by increased LC3A/B-II expression in U251MG and G34. Sim: simvastatin. All values are the mean±SD of three experiments. ^*^, *P*<0.05; ^**^, *P*<0.01. ^***^, *P*<0.001. ^****^, *P*<0.0001.

Migration and invasion are responsible for much of the malignant character of GBM and other cancers, and TGF-β is well-known to promote these behaviors through mediators such as RhoA and ROCK. In the G34 GIC line, we observed that cell spreading from adherent spheroids was completely ablated by simvastatin (Figure 3A). This prompted us to test whether the statins affected GBM cell migration and invasion. Indeed, simvastatin significantly reduced migration and invasion in U251MG (Figure 3B) and G34 cells (Figure 3C). Simvastatin induced apoptosis in U251MG and GIC G34 cells, based on increased caspase-3/7 activity (Figure 3D, left panel) and cleaved PARP expression (Figure 3D, right panel). It also caused a relative increase in the LC3A/B-II isoform, suggesting induction of autophagy in these cells (Figure 3E).

### Simvastatin effects on cancer cells are rescued by selected products of HMG CoA-reductase

**Figure 4:**
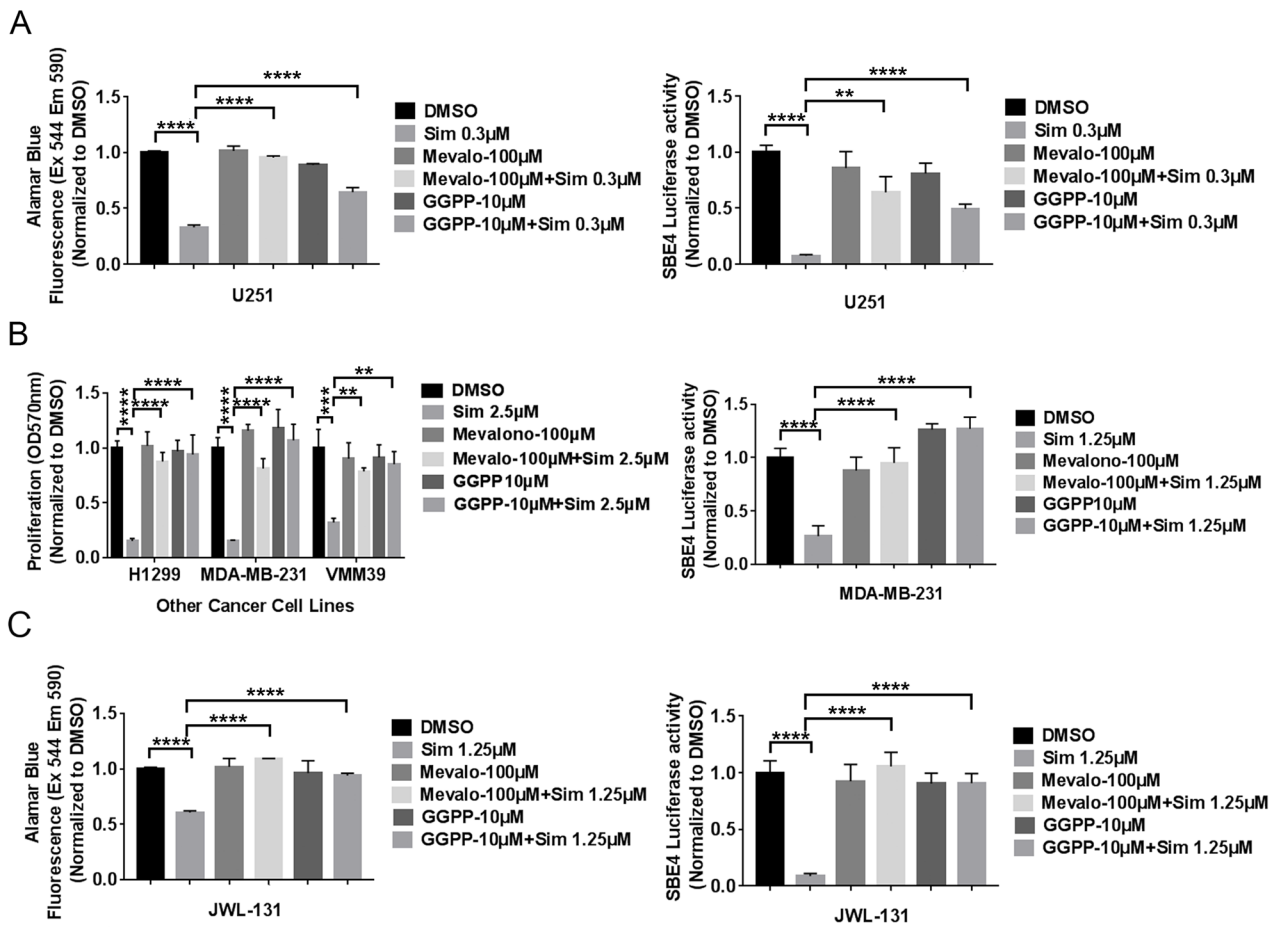
Selected exogenous products of HMG CoA-reductase rescued simvastatin-induced cell toxicity and TGF-β reporter activity in five cell lines of diverse cancer types **(A)**, **(B)**, **(C)** mevalonolactone or geranylgeranyl-pyrophosphate (GGPP) rescue of decreased viability and TGF-β activity from simvastatin in U251MG (A), other cancer cells (B), JWL-131 (C). Sim: Simvastatin. Mevalo: mevalonolactone. All values are the mean±SD of three experiments. ^**^, *P*<0.01. ^***^, *P*<0.001. ^****^, *P*<0.0001.

To verify that the statin effects on cancer cell viability and TGF-β activity are due to inhibition of HMG CoA-reductase, we assessed whether statin effects could be rescued by exogenous products and mediators of this enzyme. HMG CoA-reductase is important not only for cholesterol production, but also for regulation of prenylation—both farnesylation and geranylgeranylation. We noted complete rescue of decreased cell viability and TGF-β activity from simvastatin with exogenous mevalonolactone at 100μM or geranylgeranyl-pyrophosphate (GGPP) at 10μM concentration in GBM (Figure 4A) and other cancer cells (Figure 4B). We initially did not observe rescue of decreased viability and TGF-β activity from simvastatin with exogenous mevalonolactone or GGPP at the same concentration in GICs (data not shown). However, we noted that Tanaka et al. have reported that the experimental use of these compounds is limited by their membrane impermeability [23], leading us to hypothesize that factors in serum might promote their cellular entry and explain the lack of rescue in GICs cultured without serum. When we added 1% FBS into completed culture medium for GICs, we noted complete rescue of decreased viability and TGF-β activity from simvastatin with exogenous mevalonolactone at 100μM or geranylgeranyl-pyrophosphate (GGPP) at 10μM concentration in GIC line JWL-131 (Figure 4C). It is thus possible that bovine serum albumin or other factors in 1% serum act as carriers to help these compounds penetrate cells. Interestingly, farnesyl-pyrophosphate was not able to rescue statin effects in five diverse cancer lines (data not shown), suggesting that geranylgeranylation may be a more significant mediator in this setting.

### *In vivo* efficacy and TGF-β inhibition, apoptosis, autophagy, and prenylation inhibition by a statin

**Figure 5:**
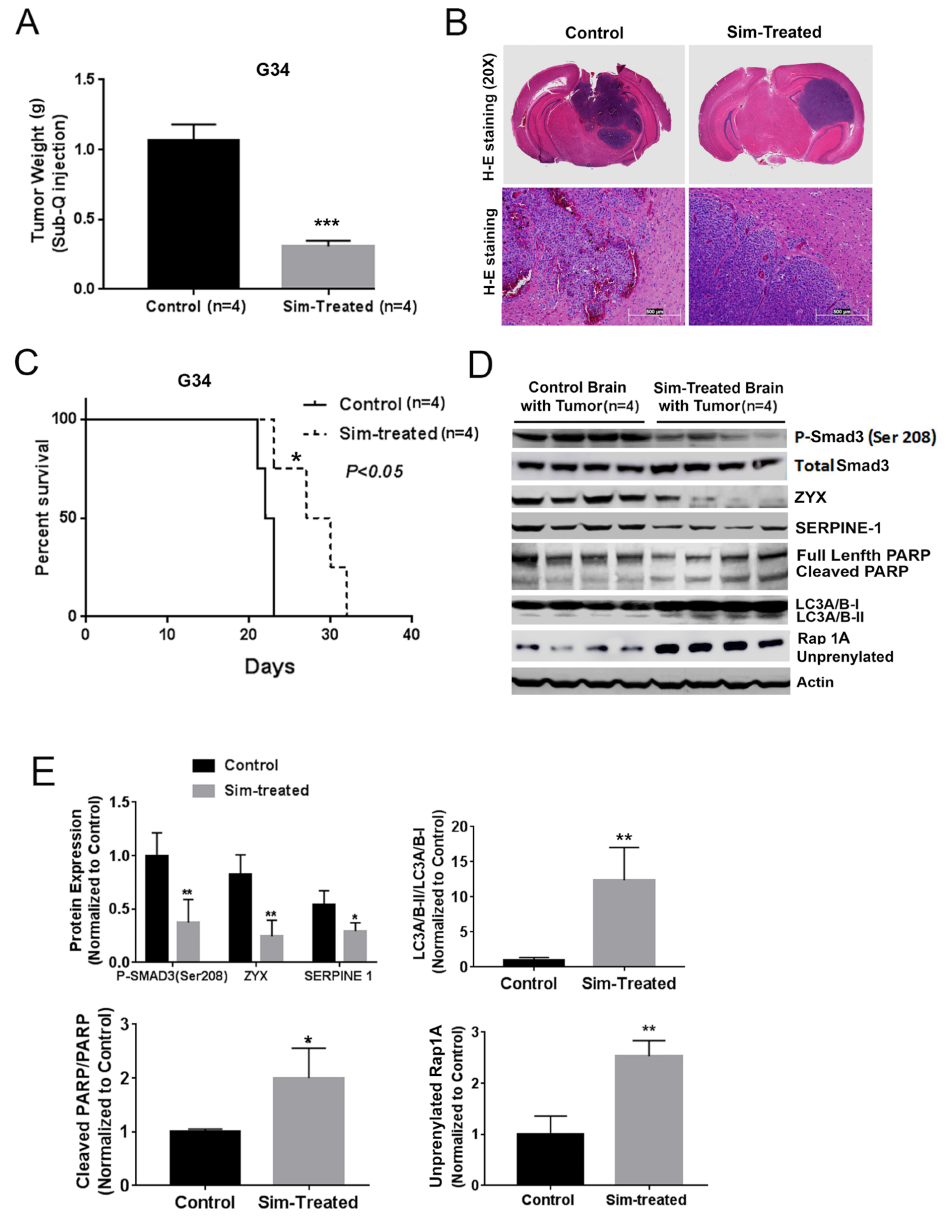
Simvastatin prolonged survival, inhibited TGF-β signaling and prenylation, and induced apoptosis and autophagy *in vivo* **(A)** Simvastatin reduced G34 tumor growth in a mouse subcutaneous model. **(B)** H+E staining of intracranial tumors from control and twice-daily simvastatin-treated (50mg/kg) group. **(C)** Twice-daily simvastatin treatment (50mg/kg) prolonged mouse survival in an intracranial G34 model. **(D)** and **(E)** Simvastatin reduced expression of the Smad3 phosphorylation at Ser-208, TGF-β target proteins ZYX and SERPINE1, increased cleaved PARP, LC3A/B-II, and unprenylated Rap1A expression in tumor-bearing brain hemispheres in the experiment from (C) as indicated by immunoblot (D) and quantification of immunoblot bands (E). All values are the mean±SD of four experiments. Sim: Simvastatin. ^*^, *P*<0.05; ^**^, *P*<0.01. ^***^, *P*<0.001.

To determine if a statin could affect GBM growth and TGF-β activity *in vivo*, we first tested simvastatin in a subcutaneous xenograft model with a mesenchymal GIC line and noted reduced tumor growth. (Figure 5A). We then investigated simvastatin effects in a GBM intracranial xenograft model, but initially did not observe efficacy of once-daily simvastatin (data not shown); the contrasting efficacy in the subcutaneous and intracranial settings suggested the possibility of a delivery issue. Notably, simvastatin is considered to have the best blood-brain barrier penetration of the statins [24], though it is reported that it is rapidly cleared from brain by p-glycoprotein and CYP3A4 pumps [25]. We therefore tested whether twice-daily simvastatin could increase the efficacy of simvastatin versus intracranial GBM, indeed noting that twice-daily simvastatin seemed to reduce invasion at the tumor margin (H+E photomicrographs in Figure 5B) and prolonged mouse survival (Figure 5C). In addition, immunoblot indicated decreased expression of the Smad3 phosphorylation at Ser-208 decreased expression of, TGF-β target proteins ZYX and SERPINE1, increased cleaved PARP, and increased LC3A/B-II and unprenylated Rap1A expression in the tumor-bearing brain of the simvastatin-treated group (Figure 5D–5E), confirming the ability of a statin to reduce TGF-β activity *in vivo*. Increases in unprenylated Rap1A on immunoblot have been used as a marker for inhibition of the prenylating enzyme Geranylgeranyltransferase I (GGTase I) downstream of statin activity [26]. We performed Kaplan-Meier analysis of ZYX and SERPINE1 versus overall survival in a GBM patient dataset (http://www.betastasis.com/glioma/rembrandt/), noting that high expression of ZYX and SERPINE1 correlated with shorter survival of patients (*P*=2.05e-4 and *P*=0.02, Supplementary Figure 4A-4B). Given that microglia and macrophages have been linked to TGF-β activity in GBMs, we also tested whether simvastatin treatment affected peritumoral macrophages/microglia; immunohistochemistry for the Iba1 marker indicated increased number and extent of Iba1+ cells in the tumor periphery with simvastatin treatment (Supplementary Figure 5A-5B).

## DISCUSSION

Our results show for the first time that the statins act as potent TGF-β inhibitors in GBM, GICs, and other cancer cells with high TGF-β activity, and that this is a dominant mechanism for their direct anti-cancer activity. We propose that these studies help clarify the role of statins in oncology, and where they might best be applied as therapeutic adjuncts. Importantly, TGF-β inhibition was observed at concentrations in the high nanomolar range—at least for high-TGF-β GIC lines—that are achievable in patients, suggesting clinical relevance. Efficacy and TGF-β inhibition were noted both *in vitro* and *in vivo*. Importantly, we also demonstrated that the statins had similar effects in other cancers, indicating that these findings are broadly applicable in oncology.

TGF-β is well-established as a driver of the epithelial-mesenchymal transition (EMT) and the mesenchymal phenotype in cancer, and there is a dire need in oncology for therapies targeting mesenchymal cancers with high TGF-β activity. The mesenchymal phenotype promotes invasiveness, metastasis, and resistance to most therapies, but its connection to TGF-β may provide a point of vulnerability. We did indeed observe a trend toward preferential activity of the statins—affecting both viability and invasiveness—in mesenchymal GIC lines, and also observed that exogenous TGF-β enhanced statin sensitivity only in high-TGF-β lines that were typically of the mesenchymal subtype. We hypothesize that exogenous TGF-β acts to boost the addiction of mesenchymal lines to TGF-β, but additional work is required to validate this proposed mechanism. They may also have higher levels of TGF-β receptors, further sensitizing them to its effects. Further studies are needed to verify the preferential activity of the statins against the mesenchymal phenotype in GBM and in other cancers. That being said, these results preliminarily indicate that the statins would best be applied as adjuncts in a regimen targeting mesenchymal cancers with high TGF-β activity; it is unlikely that they would have substantial benefit as single agents. To our knowledge, this is the first demonstration for any agent of preferential activity against cancers with high TGF-β activity.

These findings pertain to direct action of the statins on cancer cells, but TGF-β inhibition by the statins could have additional beneficial effects *in vivo*. TGF-β also helps generate a supportive tumor microenvironment, and it has been shown to suppress the anti-tumor immune response in GBM and other cancers. The statins might therefore help boost the anti-cancer immune response through TGF-β inhibition, and future studies should assess potential benefits in combining the statins with cancer immunotherapies.

From a mechanistic perspective, we implicate statin effects on Rho/ROCK phosphorylation of Smad3 as a mediator of statin TGF-β inhibition. However, it is possible that we have only partially elucidated the mechanism or mechanisms by which the statins inhibit TGF-β activity. It has previously been shown that: 1) statins can impair lipid rafts below the plasma membrane [27], and 2) these lipid rafts are important for TGF-β receptor signaling [28].

Future work will need to further clarify how best to incorporate the statins alongside current cancer therapies and those in the pipeline. With TGF-β inhibitors long anticipated and with candidates in the pipeline, it will be particularly important to test the relationship between statin use and TGF-β pathway activation in patient GBM specimens and to rigorously determine if BBB-penetrating statin use associates with a survival advantage in GBM patients.

## MATERIALS AND METHODS

### Antibodies and reagents

Smad3 (phos-S208) (Abcam, ab138659), SERPINE1 (Fisher Scientific), Rap 1 (sc-398755) and TGF-β RI Kinase inhibitor V (Santa Cruz Biotechnology), PARP (#9542S), LC3 A/B (#4108S), ZYX, and total Smad3 (Cell Signaling Technology) antibodies were obtained from commercial vendors. Simvastatin was obtained from Sigma Chemical (*in vitro* stock and *in vivo* stock for subcutaneous GIC grafts) and Selleckchem (*in vivo* stock for GIC intracranially grafted mice). TGF-β2, mevastatin, fluvastatin, lovastatin, geranylgeranyl pyrophosphate (GGPP), farnesyl pyrophosphate (FPP) and (±)-mevalonolactone were obtained from Sigma Chemical. Silencer® select pre-designed *SMAD3* siRNAs, and Silencer® Select Negative Control siRNA were purchased from Life Technology. LY2109761 was from AdooQ BioScience (A11133).

### Cell culture

U87MG, T98G, U251MG, MDA-MB-231, PC-3, and H1299 were obtained from ATCC. Melanoma cancer cells VMM39 were from Daniel Gioeli (University of Virginia). GBM cell lines were cultured in MEM media. MDA-MB-231 was in DMEM high glucose media, PC-3 in DMEM low glucose, VMM39 and H1299 in RPMI media 1640. GIC lines G34, G44 and G528 were from Jakub Godlewski (Harvard Medical School) and Ichiro Nakano (University of Alabama). GIC lines JWL-022, JWL-131, JWL-578 and JWL-592 were from Jeongwu Lee (Lerner Research Institute). GICs were cultured in Neurobasal media with N2 and B27 supplements (0.5X), with the addition of human recombinant bFGF and EGF (25ng/ml each; R&D Systems). Short tandem repeat profiling was performed within the last six months to confirm the identity of established cell lines or to confirm the human origin of GIC lines lacking established STR profiles. All cell cultures were screened negative for mycoplasma by MycoAlertTM PLUS Mycoplasma Detection Kit (Lonza).

### Cell counting, crystal violet and alamarblue cell viability assay

GBM cells were plated in 96-well plates (10^3^cells/well) in 10% serum media, then 1% serum media the next day. GICs were plated in 96-well plates (3×10^3^ cells/well) pre-coated with 0.01% Poly-L-Ornithine plus 10 μg/ml laminin mixture. Statin and commercial TGF-beta inhibitors were added to the medium for 6 days. At day 7, cells were counted on a hemocytometer two times per well. For some experiments, alamarBlue and 0.1% crystal violet were used for cell viability assay.

### Cell rescue by selected products of HMG CoA-reductase after simvastatin treatment

In 1% serum medium, 100 μM of (±)-mevalonolactone and 10 μM of GGPP and FPP were added, then after an hour simvastatin added and cell proliferation assayed at six days.

### CyQUANT® direct cell proliferation assay and Caspase-Glo® 3/7 assay

U251MG and G34 were treated with simvastatin for six days and the CyQUANT® Direct Cell Proliferation kit used per the manufacturer's instructions. The Caspase 3/7 activity assay was also used per the manufacturer's instructions. Luminescence was measured on a Promega GloMax 20/20 luminometer and normalized with fluorescence data from the CyQUANT® Direct Cell Proliferation assay.

### Preparation of whole cell extract and immunoblotting

Cultured cells were lysed in RIPA buffer containing 0.1% SDS, protease inhibitors and phosphatase inhibitor cocktail III. Proteins were solubilized in sample buffer (Invitrogen) and boiled at 70°C for 10 min. 30 μg of protein was loaded. Membranes were blocked with 5% nonfat dry milk in Tris Buffered Saline with 0.05% Tween 20 (TBST) for 1 hour, then incubated overnight at 4°C with primary antibodies in 1% bovine serum albumin in TBST and then AP-conjugated secondary antibodies (1:5000) (Sigma). Specific bands were visualized with premixed BCIP/NBT solution, photographed with ChemiDoc™ Touch Imaging System, and quantified with NIH Image J software.

### SBE-4 luciferase assay

At day 5 after plating cells +/- TGF-β2, PRL-Renilla and SBE-4 luciferase plasmids (with 4 SMAD-Binding Elements upstream of promoter and firefly luciferase, from the Vogelstein laboratory) were transfected with Fugene 6 for two days per the manufacturer's instructions. The SBE4-luciferase plasmid is a well-established and accurate indicator of canonical TGF-β activity through Smad3 [29]. Cells were assayed with the Dual-Luciferase Reporter assay system kit (Promega). Luminescence was measured on a Promega GloMax 20/20 luminometer. Results were normalized by dividing by signal from PRL Renilla luciferase vector.

### RhoA G-LISA activation assay

U251MG in 1% serum medium or G34 (made adherent with 0.01% Poly-L-Ornithine plus 10 μg/ml laminin) were treated with simvastatin and at day 7 assayed with the RhoA G-LISA Activation Assay Kit (Cytoskeleton, Inc.) per the manufacturer's protocol.

### *SMAD3* siRNA transfection

GBM, GICs and other cancer cells were transfected with Silencer® select pre-designed *SMAD3* siRNAs and Silencer® Select Negative Control siRNAs with Lipofectamine RNAiMAX per the manufacturer's guidelines, then at day 3 treated with a single dose of simvastatin and at day 5 harvested for cell counting or crystal violet assay. All data were normalized to Silencer® Select Negative Control siRNAs. For SBE4 luciferase assay, at day 4 cells were transfected with SBE4 and PRL Renilla luciferase plasmids and two days later luminescence measured as described above.

### Cell migration and collagen type IV invasion assay

Trans-well inserts with 8-μm pores were coated with 60 μl of collagen type IV (0.125 mg/ml) for cell invasion assay and non -coated inserts used for cell migration assay. Cell suspensions with or without simvastatin (0.5 × 10^5^ cells/insert) in serum-free media were added to the upper chamber, 500 μl of cultured media with 2% fetal bovine serum to the lower chamber, and simvastatin added an hour later. After 18 hours, cells on the upper membrane surface were gently scraped away and adherent cells on the lower surface stained with 0.1% crystal violet. Five photomicrographs were counted for each group.

### *In vivo* studies

Mouse studies were performed with a University of Virginia IACUC-approved protocol. For the subcutaneous tumor experiment, 1 × 10^6^ G34 cells were engrafted at the flanks athymic Nu/nu mice and after 3 days of tumor growth, mice with similar tumor sizes began treatment with oral gavage of 50 microliters of simvastatin 50 mg/kg/day (n=4) or equal volume vehicle (n=4) (0.5% methylcellulose) twice per day. Tumor measurements were done every day and mice euthanized after tumors reached a size of 1 cm or showed necrosis. Tumors were dissected and weighed.

Eight-week-old female BALB/c SCID NCr (from NCI) mice were injected intracranially with human GICs G34 (500 cells) in 5 μl of Hank's Buffered Saline Solution into the right caudate/putamen. At 6 days post-implantation, treatment began with 50 μl simvastatin (50 mg/kg/day, n=5) or vehicle oral gavage (n=5) twice per day. Pre-moribund mice were euthanized and tumor-bearing brains frozen for immunoblot.

### Statistics

Hypergeometric tests were performed with online calculators to determine enrichment of statins among drugs with cancer cytotoxicity significantly correlating with *SERPINE1* and *ZYX* expression in the CellMiner database. Statistical analyses were performed using GraphPad Prism 7 (GraphPad Software, Inc.). The significance of differences between two groups was tested by t-test. Tukey's multiple comparisons test of one-way ANOVA was used to analyze more than two groups. XY graph and correlation of XY analyses were used to assess correlations of statin sensitivity IC50 to SBE4 luciferase activity. Kaplan-Meier plots and log-rank analysis were used for survival studies.

## SUPPLEMENTARY MATERIALS FIGURES AND TABLES



## Abbreviations

GBM: glioblastoma
GIC: GBM-initiating cell
TGF-β: Transforming Growth Factor-beta
SBE4 luciferase reporter: 4 SMAD-Binding Elements upstream of promoter
GGPP: geranylgeranyl-pyrophosphate
Sim: Simvastatin
Mevalo: mevalonolactone;
