# Supplementary material for: Statins affect human glioblastoma and other cancers through TGF-β inhibition
Source: Oncotarget. 2019 Mar 1;10(18):1716–28. doi: 10.18632/oncotarget.26733 (PMC6422202; doi:10.18632/oncotarget.26733)
Supplement: Supplementary file 1 [file oncotarget-10-1716-s001.pdf]

Statins affect human glioblastoma and other cancers through TGF-β inhibition

SUPPLEMENTARY METHODS

Soft agar colony formation

A total of 5×10^3 GBM cells or G34 cells per well were seeded in a 48-well plate in 0.3 ml of 0.4% agar medium over 0.5 ml of 0.8% agar Phosphate-buffered saline (PBS). Medium (1 ml) was added after plating the top agar for one hour. Simvastatin was added to the top agar and medium with or without TGF-β2 at the desired concentration. After 28 days, media were carefully removed and 0.5ml of 0.005% crystal violet was added overnight. Photomicrographs of colonies were taken at 50x magnification. The number of colonies was determined with ImagePro 4.5.

High-throughput screening of a natural product library for targeting a mesenchymal GIC line (Supplementary Figure 2 experiment):

Mesenchymal GBM cell line GBM1123 was cultured in DMEM/F12 with 1% penicillin-streptomycin, GlutaMAX (1X), B27 (1X), heparin (5μg/ml), bFGF (20ng/ml) and EGF (50ng/ml) for sphere culture or DMEM/F12 with 10% serum and 1% penicillin-streptomycin for adherent culture. GBM1123 neurospheres were seeded in a 384-well plate at 1.0×10^3 cells/well) in serum-free media and incubated for 72h. Screening samples composed of 28,160 microbial metabolites (Technology Research Association for Next generation natural products chemistry) were added to neurospheres at a concentration of 0.5% and incubated for another 72h. Neurospheres were then stained with 1μg/ml propidium iodide (PI) and 1μg/ml Hoechst33342 (Dojindo) for 1h. Fluorescent images were observed using IN Cell Analyzer 6000 (GE Healthcare) or Opera Phenix (PerkinElmer).

Adherent cultures of GBM1123 in serum media were seeded at 1.0×10^3 cells/well in 384-well microplates (Greiner) and incubated for 4h. The same screening samples (0.5%) were added and incubated for 72h. Cell viabilities were measured using WST-8-based Cell Counting Kit8 (Dojindo) and Envision multilabel plate reader (PerkinElmer). Statins from US-Drug Collection (MicroSource) and Chemical library (Prestwick) were used for secondary evaluation.

Immunohistochemical staining of simvastatin- or control-treated brains with GBMs for Iba1 to indicate macrophages/microglia

The tissue sections were deparaffinized, dehydrated, antigen unmasked, and blocked. Then rabbit anti-mouse Iba 1 primary antibody (Invitrogen, 1:500) was applied and incubated at 4°C overnight. Biotinylated donkey anti-rabbit (Jackson ImmunoResearch Laboratories) antibody was used as secondary antibody. After incubation with an avidin-biotin complex (VECTOR LABORATORIES, INC.) at room temperature for 30 mins, immunoreactivity was visualized by incubating the sections with 3, 3-diaminobenzidine tetrahydrochloride (Dako Corp) to produce a brown precipitate and then counterstained with hematoxylin. Photos were taken with a Nikon microscope equipped with a CCD camera.

Iba1 immunostainings were quantified using the Image J program. Color threshold was selected within the image/adjust menu. An appropriate color threshold (RGB) was chosen (Red: 0-103, Green: 0-104, Blue: 0-69) to include the staining of signal ($Iba1^+$). The area outside of tumor was manually selected. Under the Analyze menu, Measure was selected for obtaining the number within the selected area. Within the Analyze/Analyze particle menu, Size was set from 0.01 to infinity, Show was selected to Masks to ensure non-specific staining was excluded and Summarize was selected to obtain the count of staining cells and %Area (total area with signal divide by selected outside of tumor area). %Area and normalized count of staining cells (count of staining cells divided by selected area) from each group were then used for statistical analysis.

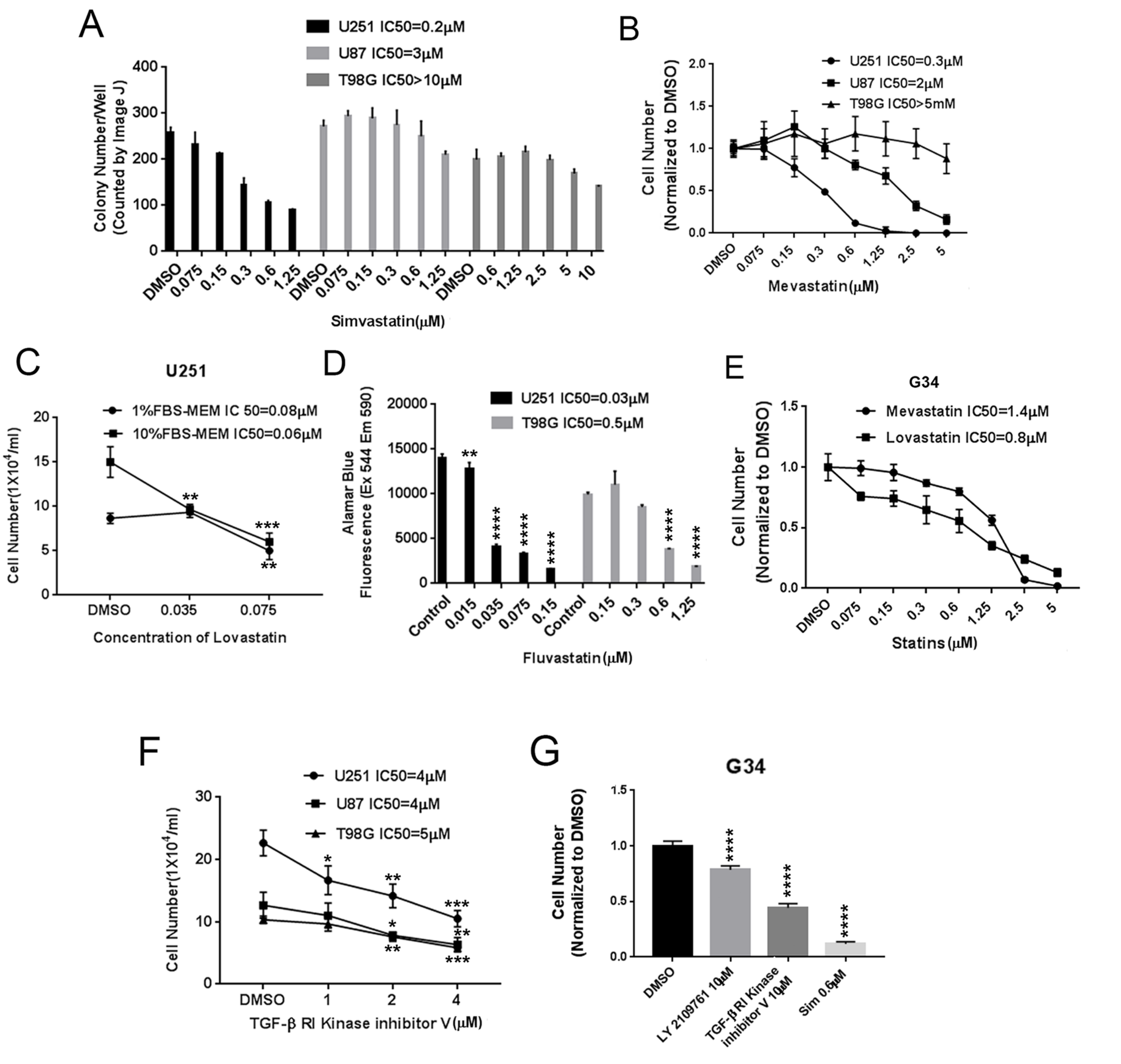

Supplementary Figure 1: Statins and commercially available TGF-β inhibitors reduce GBM cells and GICs viability. **(A)** IC50 (μM) values of simvastatin for U251MG, U87MG, and T98G cells in soft agar with 10%FBS-MEM medium for 30 days. **(B)** IC50 (μM) values of mevastatin for U251MG, U87MG, and T98G cells with 1%FBS-MEM medium for 6 days. **(C)** Increasing single doses of lovastatin reduced proliferation of U251 cells in 1% and 10% FBS medium conditions for 6 days. **(D)** Variation of IC50 (μM) values of lovastatin with increasing single doses for U251 and T98G cells with 1% FBS medium condition for 6 days. **(E)** Cell counts of lovastatin- and mevastatin-treated G34 GICs. **(F)** Cell counts of U251MG, U87MG and T98 GBM cells exposed to existing TGF-β inhibitors TGF-β RI Kinase inhibitor V in 1%FBS medium. **(G)** The effects of TGF-β RI Kinase inhibitor V, LY2109761 and simvastatin on cell counts in G34 cells. *, $P<0.05$. **, $P<0.01$. ***, $P<0.001$. ****, $P<0.0001$.

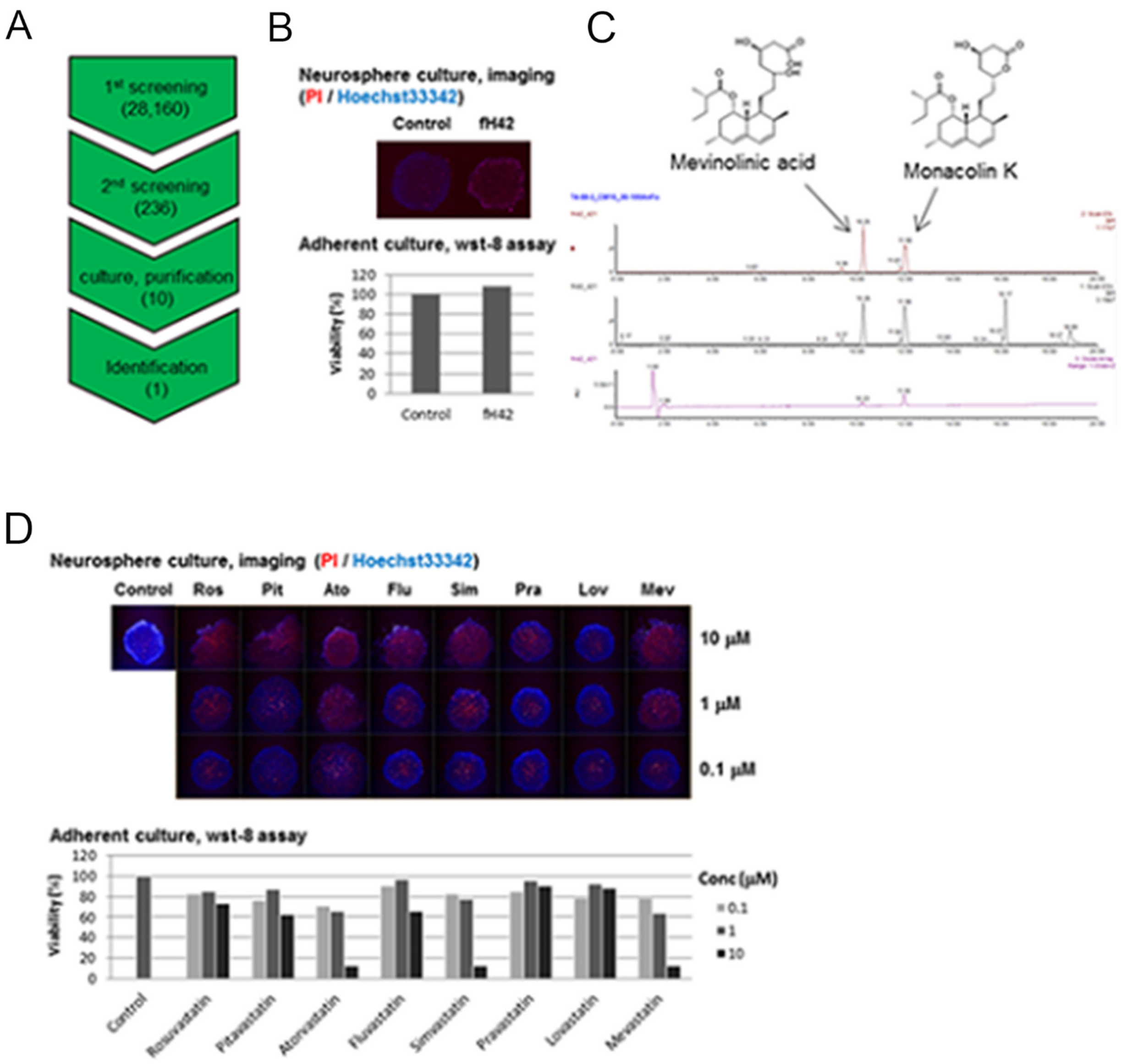

Supplementary Figure 2: High-throughput screening of a natural product library for targeting the mesenchymal GSC line GBM1123. **(A)** Step sequences indicated that 28,160 samples tested in the 1st screening produced 236 hits that have selective toxicity to neurospheres without toxicity to adherent cells. From the 2nd screening, 10 potent hits which had strong and dose-dependent activity were selected. Microbial strains originating from these hit samples were cultured and their metabolites analyzed to identify the active component. Active components from only 1 strain were succeeded to be identified. **(B)** Metabolite of the fH42 fungal strain showed selective toxicity to neurospheres with increased PI intensity. **(C)** From the fH42 metabolite, mevinolinic acid and mevinolin (also known as monacolin K or lovastatin) were purified using HPLC as active components. **(D)** All 8 statin drugs from a chemical library were evaluated.

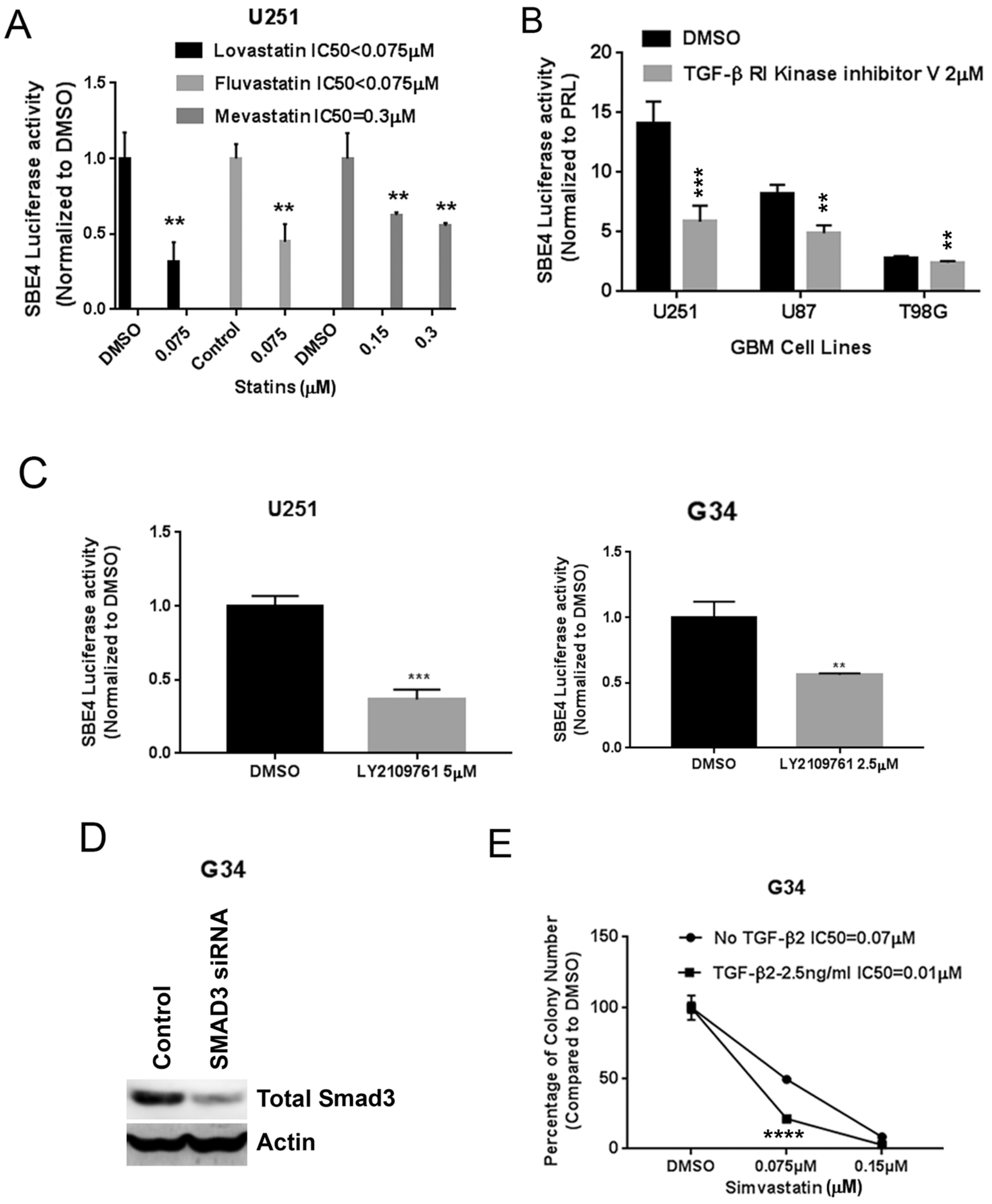

Supplementary Figure 3: Statins and commercially available TGF-β inhibitors effects on TGF-β reporter activity in GBM cells and G34 GICs. **(A)** Lovastatin, fluvastatin, and mevastatin reduced SBE4-luciferase activity in U251. **(B)** Normalized SBE4 luciferase reporter activity in U251MG, U87MG, and T98G GBM cells with daily dosing with 2μM of TGF-β RI Kinase inhibitor V for 6 days. **(C)** Normalized SBE-4 luciferase reporter activity in U251 and G34 GICs exposed to TGF-β inhibitor LY2109761 for 6 days. **(D)** *SMAD3* siRNA knockdown reduced total Smad3 protein expression. (**E**) Colony number of G34 GICs in soft agar after 30 days of simvastatin +/-exogenous TGF-β. **, $P<0.01$. ***, $P<0.001$. ****, $P<0.0001$.

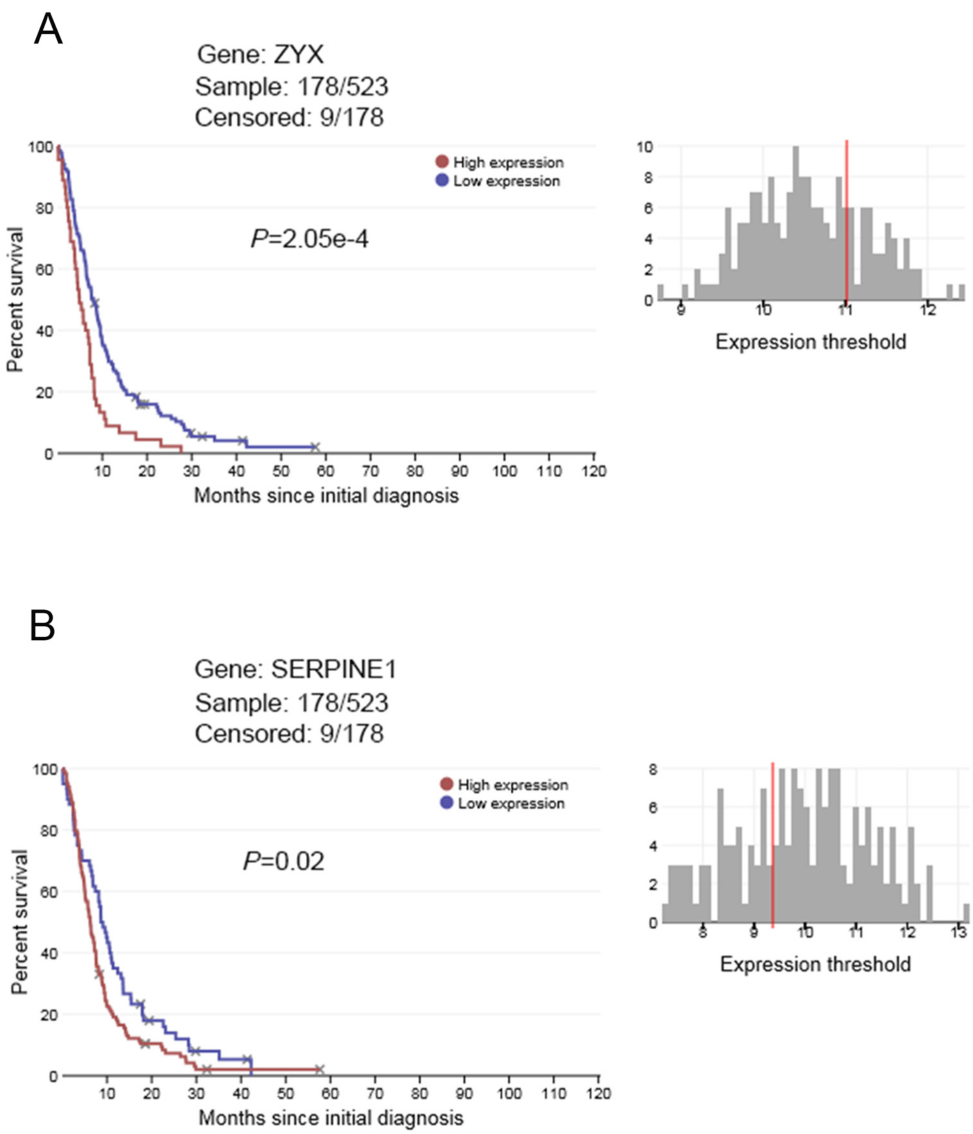

Supplementary Figure 4: Kaplan-Meier survival analysis. **(A)** Correlation of ZYX expression with overall survival of GBM patients. **(B)** Correlation of SERPINE1 expression with overall survival of GBM patients.

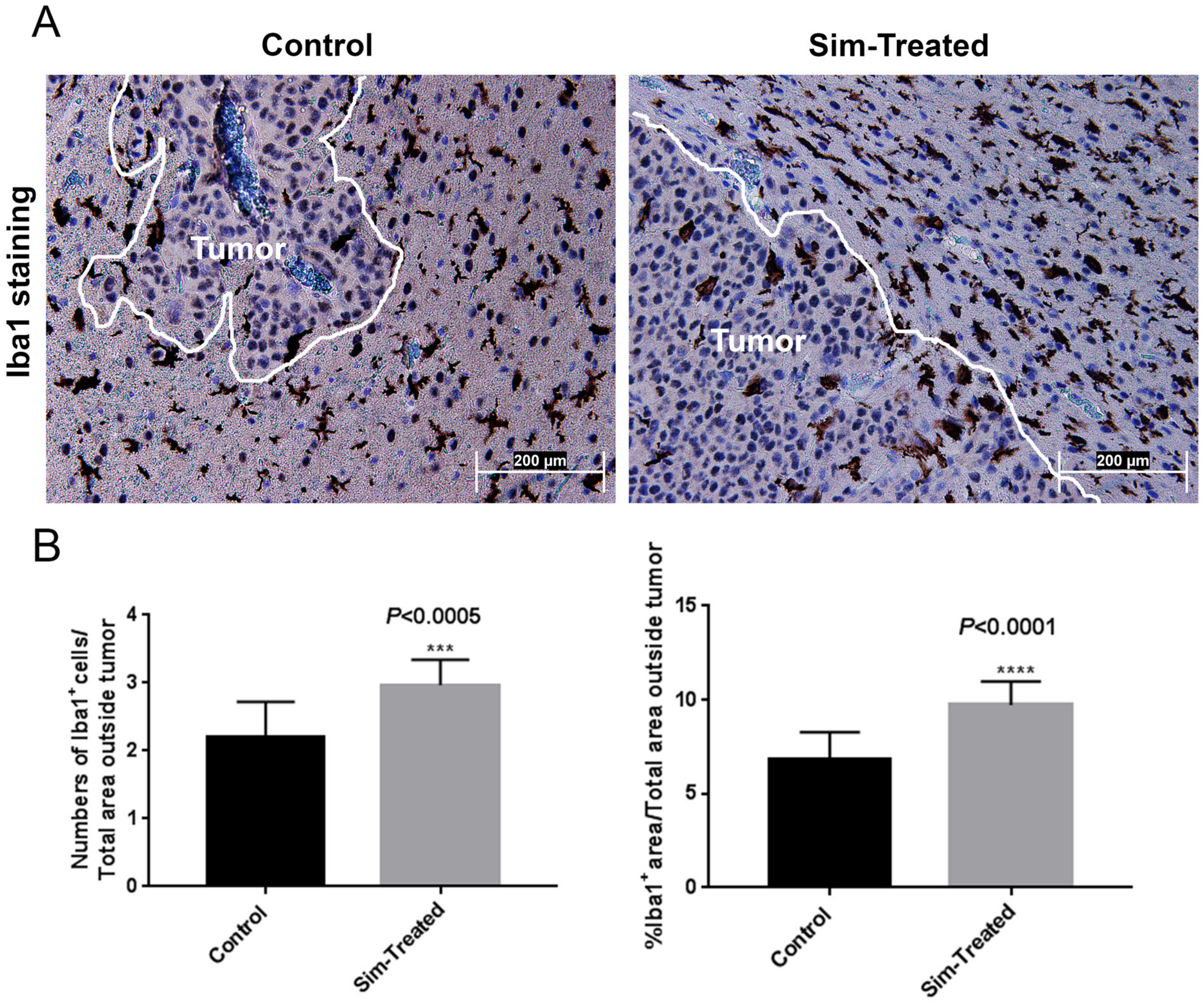

Supplementary Figure 5: Simvastatin increases peritumoral macrophages/microglia. **(A)** and **(B)** Simvastatin (50mg/kg) treatment increased the number and extent of Iba1+ macrophages/microglia outside tumor.
